# Deep sequencing of small RNA libraries reveals dynamic regulation of conserved and novel microRNAs and microRNA-stars during silkworm development

**DOI:** 10.1186/1471-2164-11-52

**Published:** 2010-01-20

**Authors:** Guru Jagadeeswaran, Yun Zheng, Niranji Sumathipala, Haobo Jiang, Estela L Arrese, Jose L Soulages, Weixiong Zhang, Ramanjulu Sunkar

**Affiliations:** 1 Department of Biochemistry and Molecular Biology, Oklahoma State University, Stillwater, OK 74078, USA; 2 Department of Computer Science and Engineering, Washington University in St. Louis, St Louis MO 63130, USA; 3 Department of Entomology and Plant Pathology, Oklahoma State University, Stillwater, OK 74078, USA; 4 Department of Genetics, Washington University School of Medicine, St. Louis, MO 63110, USA

## Abstract

**Background:**

In eukaryotes, microRNAs (miRNAs) have emerged as critical regulators of gene expression. The Silkworm (*Bombyx mori *L.) is one of the most suitable lepidopteran insects for studying the molecular aspects of metamorphosis because of its large size, availability of mutants and genome sequence. Besides, this insect also has been amply studied from a physiological and biochemical perspective. Deep sequencing of small RNAs isolated from different stages of silkworm is a powerful tool not only for measuring the changes in miRNA profile but also for discovering novel miRNAs.

**Results:**

We generated small RNA libraries from feeding larvae, spinning larvae, pupae and adults of *B. mori *and obtained ~2.5 million reads of 18-30 nt. Sequence analysis identified 14 novel and 101 conserved miRNAs. Most novel miRNAs are preferentially expressed in pupae, whereas more than 95% of the conserved miRNAs are dynamically regulated during different developmental stages. Remarkably, the miRNA-star (miR*) of four miRNAs are expressed at much higher levels than their corresponding miRNAs, and their expression profiles are distinct from their corresponding miRNA profiles during different developmental stages. Additionally, we detected two antisense miRNA loci (miR-263-S and miR-263-AS; miR-306-S and miR-306-AS) that are expressed in sense and antisense directions. Interestingly, miR-263 and miR-306 are preferentially and abundantly expressed in pupae and adults, respectively.

**Conclusions:**

We identified 101 homologs of conserved miRNAs, 14 species-specific and two antisense miRNAs in the silkworm. Our results provided deeper insights into changes in conserved and novel miRNA and miRNA* accumulation during development.

## Background

Transcriptional regulation alone is insufficient to ensure tight control of gene expression in specific cells or tissues. Recently discovered microRNA (miRNA)-directed post-transcriptional regulation can provide an efficient fine-tuning of target gene expression in certain cell or tissue types and, thus, coordinate the spatial and temporal control [1,2]. Furthermore, miRNA-guided suppression of the target genes can be quickly relieved, and such reactivation is faster than transcriptional activation of a genomic locus; thus, miRNAs can act as reversible regulators [1]. Because of the versatility, miRNAs have evolved as a major class of gene-regulatory molecules critical for diverse biological processes such as cell proliferation, differentiation, apoptosis, stress response, tumorigenesis, diabetes and heart failure in eukaryotes [3-11].

In animals, most miRNA genes are transcribed by RNA polymerase II, yielding transcripts called primary miRNAs (pri-miRNAs), which are initially processed by a complex containing Drosha and then by Dicer-1 to excise miRNA:miRNA-star (miR:miR*) duplexes. One strand of the duplex (miR) is more stable and preferentially incorporated into an RNA-induced silencing complex (RISC). The miRNA then guides the RISC to regions of complementarity in the target site, where it downregulates the gene expression, often by blocking protein production or by degrading the target mRNA [12-16].

Insect metamorphosis is a complex, highly conserved, and strictly regulated process of developmental events. During metamorphosis, diverse morphological, physiological, biochemical and molecular events result in distinct changes such as cell proliferation, programmed cell death, tissue remodeling and cell migration [17]. The silkworm is an ideal model for studying metamorphosis in holometabolous insects, because of its large size, the availability of mutants with nearly fully sequenced genome. Additionally, this insect has been amply studied from a physiological and biochemical perspective [18]. Many agricultural pests belonging to Lepidoptera cause economic damage to commercial crops. Thus, molecular studies focusing on silkworm metamorphosis should provide better understanding of insect gene regulation and novel targets for pest control.

Thus far, miRNAs cataloging in insects is primarily carried out in *Drosophilid *species, and many miRNAs were discovered both by direct cloning [19-23] and by bioinformatic methods [24,25]. Besides *Drosophila*, mosquito (*A.gambiae*) [26,27], honey bees (*A.mellifera*) [28,29], red flour beetle [30] and locusts [31] are some of the insect species, in which miRNAs has been identified. Most existing studies of small RNAs in the silkworm have focused on identification of miRNAs using computational strategies [32-35]. Such studies can identify conserved miRNAs but not species-specific ones. Recently cloning of miRNAs in the silkworm has been reported [36], in which Zhang and co-authors analyzed 95,184 unique small RNA reads and annotated 354 of them as miRNAs; none were based on miR* sequences. Surprisingly, 253 of these reported miRNAs (>70%) were represented by single reads, and an additional 51 miRNAs were represented by two reads in the library. One of the most important criteria for annotating novel miRNAs is cloning their miR* sequences and this becomes even more important for annotating species-specific miRNAs. We have recovered most of the sequences (>90%) reported by Zhang et al., [23] multiple times in multiple libraries, yet could not annotate them as miRNAs due to the lack of miR* support. Furthermore, Zhang et al. [36] did not measure changes in miRNA abundance at different developmental stages, because RNA from various stages was pooled for library construction in their study.

Deep sequencing of small RNAs can be used to reliably measure modest changes in miRNA abundance among different samples; such changes are unlikely to be identified by sequencing low numbers of clones (i.e., traditional small RNA library sequencing) or hybridization-based methods such as small RNA blot and miRNA array analyses. The deep sequencing study is also well suited for the discovery of species-specific miRNAs expressed at low abundance. Indeed, in this study, by using deep sequencing we uncovered 15 novel miRNAs that appear to be silkworm-specific. Of the 101 conserved miRNAs identified in this study, most are dynamically regulated during different developmental stages of *B. mori*. Interestingly, the abundance of miR* species of several conserved miRNAs was greater than that of their miRNAs, and their levels varied greatly at various stages. We also discovered two antisense miRNAs in the silkworm. This study provides deeper insights into dynamic regulation of the conserved and novel miRNA and miRNA* levels during different developmental stages and suggests that both universal and silkworm-specific miRNAs play important roles in expression regulation.

## Results

**Figure 1:**
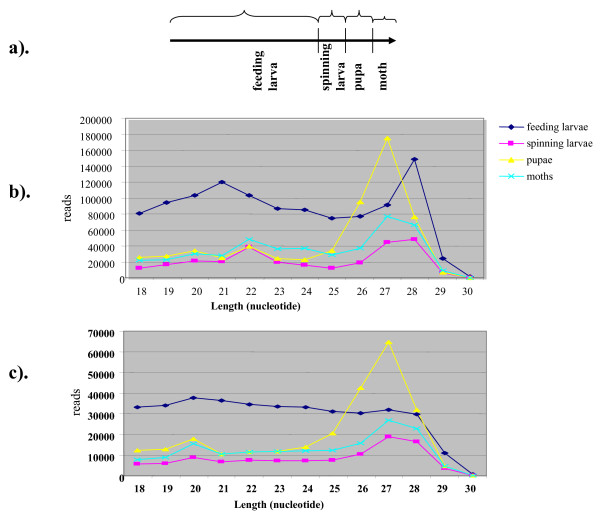
**Length distribution and abundance of small RNAs in silkworm**. a) Schematic representation of the silkworm life cycle beginning with the feeding larval stage. Abundance of each size class of small RNAs based on nucleotide (nt) length in feeding larvae, spinning larvae, pupae and moths plotted using total small RNA reads (b) and unique reads (c).

Insect metamorphosis is a highly complex and integrated set of developmental processes coordinated by the action of hundreds of genes [17]. Because miRNAs are important gene regulators, we monitored the changes in miRNA expression during larval-pupal, and adult stages of the silkworm. We constructed two small RNA libraries, one from feeding larvae (4^th ^and 5^th ^instars) and the other from spinning larvae (Figure 1a). Insects from these two stages differ significantly in physiology. 4^th ^and 5^th ^instar larvae are voracious eaters, whereas spinning larvae do not consume food, instead, they oozes silk and spin cocoon. Besides, the usage of spinning larvae may help to identify transiently regulated miRNAs (consequently, miRNA-mediated processes) during the larval-to-pupal molt. We constructed two additional libraries, from pupae and moths (Figure 1a). Pupae undergo complex histolysis and histogenesis, and their physiology and metabolism are different from that of larvae and adults. The adult library may reveal information on miRNA-mediated regulation of reproduction. While constructing the libraries, we used a bar-coded 5' adapter for each of the four RNA samples. This helped us sequence these libraries together to a reasonable depth using the sequencing-by-synthesis (SBS) sequencing technology (Illumina inc.), yet the sequences can be traced back to their original stages.

A dataset of about 2.5 million reads (1 147 753, 599 279, 289 303 and 463 800 reads from the feeding larvae, spinning larvae, pupae, and moths, respectively) ranging from 18 to 30 nt was obtained after trimming the adapter sequences. A significant number of reads (~300,000) lacking the bar-code sequences were eliminated from the analysis because these could not be assigned to the source libraries. From the size distribution of total reads, we found a distinct bimodal distribution, with a peak around 20-22 nt representing miRNAs and another distinct peak around 26-29 nt mostly representing longer piRNA-like small RNAs in all four stages (Figure 1b and 1c). Size-dependent bimodal distribution of small RNAs was also evident, with unique small RNAs plotted from spinning larvae, pupae and moths but not from feeding larvae (Figure 1b and 1c). A peak for the 26 to 29-nt size class with total reads but not unique reads specifically in feeding larvae suggests that these small RNAs are more diverse in larvae and thus not enriched for unique small RNAs. Overall, the peak represented by the 26- to 29-nt size class was greater than that for the 20- to 22-nt size class and suggested that the longer-sized small RNAs are more abundant in all four stages examined. Notably, the peak represented by the long (26-29 nt) unique small RNAs was much greater in the pupal stage (Figure 1c). The greater abundance of unique small RNAs of long size class in pupae implies a critical role for these small RNAs in the pupal stage.

Of 2.5 million total reads, 1,581,810 could be mapped to the available silkworm genome. We used the latest version of silkDB v2.0 to map and analyze these small RNAs. After eliminating redundant sequences from the dataset, only unique sequences represented by 739,119 reads were analyzed further (Table 1). Subsequent sequence analysis eliminated reads corresponding to rRNAs, tRNAs, snRNAs and snoRNAs and repetitive elements, assuming that these are degradation products [37-39]. The unique dataset with read counts was used to identify conserved and novel miRNAs in silkworm.

**Table 1:** Sequence analysis of four different small RNA libraries

	Feeding larvae	Spinning larvae	Pupae	Moth	Total
non-codingRNAs (rRNA, tRNAs etc.,)	558345	167369	94118	169508	989340
conserved and novel miRNAs	43278	70143	67256	69913	250590
messenger RNAs	101508	27736	9577	13564	152385
mitochondrial RNAs	7356	951	231	201	8739
repetitive elements	147327	40034	16522	30965	234848
subtotal	832966	293424	182808	272612	1581810
cannot be mapped to the available genome	314787	305855	106495	191188	918325
Total	1147753	599279	289303	463800	2500135
Unique small RNAs	350095	245339	101669	150197	739119

### Novel miRNAs in silkworm

**Figure 2:**
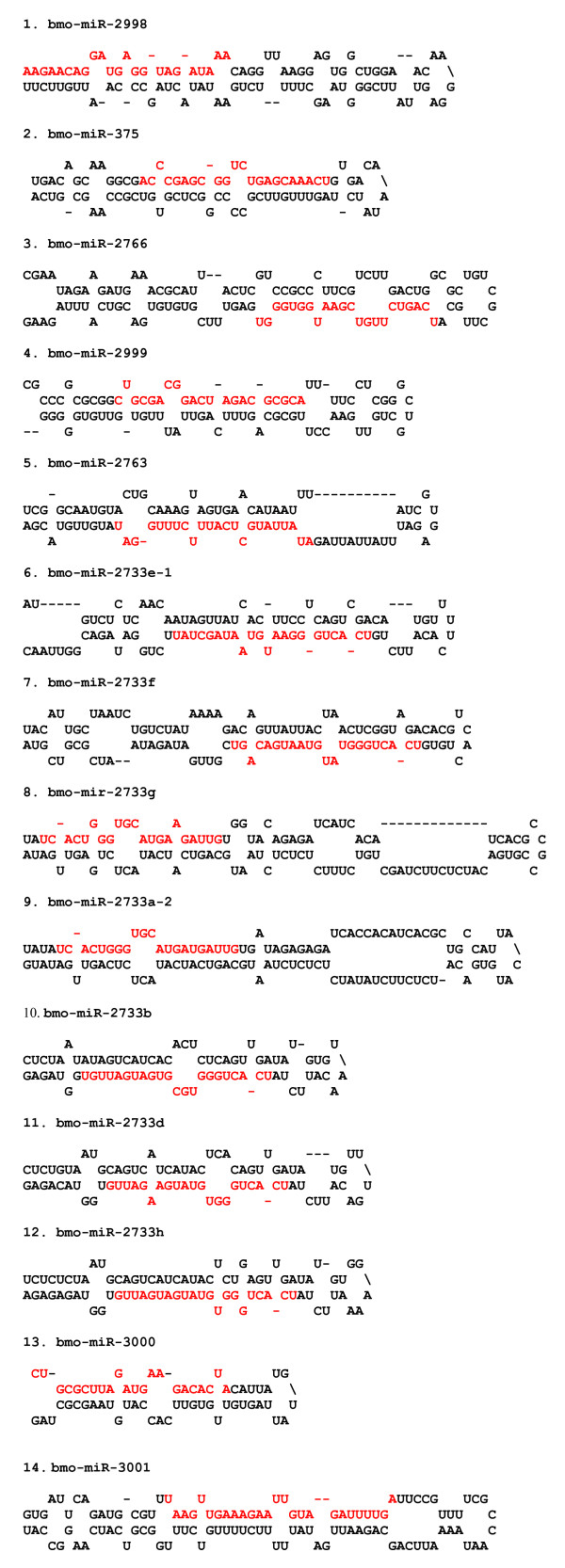
**Predicted fold-back structures for the newly identified miRNAs in silkworm**. Mature miRNA sequence is shown in red letters.

The appearance of both the miRNA and its corresponding miR* in a dataset would provide compelling evidence for annotation of a novel miRNA. Accordingly, we have annotated 14 small RNAs as novel miRNAs in the silkworm by identifying both miR and miR* sequences in our libraries (Table 2). Using their precursor sequences, we were able to predict fold-back structures for these novel miRNAs (Figure 2). miR* sequences were not represented in our dataset for two small RNAs (bmo-mir-2733e-1 and bmo-mir-2733f) but were annotated as novel miRNAs because these belong to a novel miRNA family with seven members (Table 2). Additionally, we recovered several dozens of unique small RNAs that resemble miRNAs on the basis of sequence characteristics (begin with 'U', 20-23 nt long, and with a predicted fold-back structure for their precursors). Because their miR* sequences were not detected, these were not annotated as miRNAs for now. Retrieval of relatively high number of reads from multiple libraries and appearance of miR*, coupled with predicted fold-back structure for the precursor, strongly supported that these mature miRNAs are processed from hairpin structures in the silkworm. Since, we were unable to find homologs for these miRNAs in related insects or other animal species; we annotated them as species-specific miRNAs.

**Table 2:** Identification of novel silkworm-specific miRNAs based on sequencing miRNAs and miRNA_Stars in different developmental stages.

miRNA	Sequence of mature miRNA	Feeding larvae		Spinning larvae		Pupae		Moths		Remarks
		miR	miR*	miR	miR*	miR	miR*	miR	miR*	
bmo-mir-2998	AAGAACAGGAUGAGGUAGAUAAA	26	1	9	0	10	0	88	1	
bmo-mir-375	ACCCGAGCGGUCUGAGCAAACU	30	6	4	2	25	11	11	3	
bmo-mir-2766	UCAGUCUUGUCGAAUGGUGGGU	2205	284	2657	166	4588	341	2328	133	
bmo-mir-2999	CUGCGACGGACUAGACGCGCA	20	2	3	0	16	0	107	0	
bmo-mir-2763	AUAUUAUGCUCAUUUCUUUGGAU	15	0	49	0	39	1	199	0	
bmo-mir-2733e-1	UCACUGGGAAUGUAAUAGCUAU	1	0	4	0	273	0	1	0	Family
bmo-mir-2733f	UCACUGGGUAUGUAAUGACAGU	1	0	0	0	83	0	1	0	Family
bmo-mir-2733 g	UCACUGGGUGCAUGAAGAUUG	2	0	0	0	629	2	1	0	Family
bmo-mir-2733a-2	UCACUGGGUGCAUGAUGAUUG	16	0	6	0	2718	2	93	0	Family
bmo-mir-2733b	UCACUGGGUGCGUGAUGAUUGU	0	0	0	0	134	3	0	0	Family
bmo-mir-2733d	UCACUGGGUGUAUGAAGAUUG	0	0	2	0	392	1	5	0	Family
bmo-mir-2733 h	UCACUGGGUGUAUGAUGAUUG	0	0	1	0	163	2	1	0	Family
bmo-mir-3000	CUGCGCUUAGAUGAAGACACUA	53	1	7	0	6	0	6	0	
bmo-mir-3001	UAAGUUGAAAGAAUUGUAGAUUUUGA	4	7	7	8	12	2	14	14	

**Figure 3:**
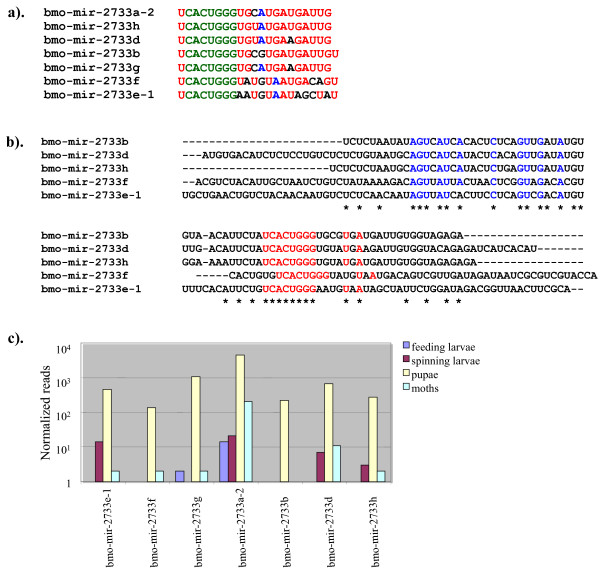
**Identification of a novel miRNA family**. (a) sequence alignment of the mature miRNAs, (b), sequence alignment of the miRNA precursor (miRNA and miRNA* are shown in red and blue colored letters, respectively) and c), expression levels of the individual members in different developmental stages.

Sequence alignment indicated that a new miRNA family represented by seven members (bmo-mir-2733a-2, bmo-mir-2733b, bmo-mir-2733d, bmo-mir-2733e-1, bmo-mir-2733f, bmo-mir-2733 g, and bmo-mir-2733 h) was identified in this study (Figure 3a). The seed region (2-8 nt), which is critical for target recognition, is identical in all these seven members, and the sequence conservation extends beyond the seed region. Furthermore, the miR*, for five of them, is also highly conserved (Figure 3b). The miRNAs, bmo-mir-2733a-2 and bmo-mir-2733 g do not display sequence conservation except in the mature miRNA region. These two miRNAs also display differences with respect to miRNA organization within the hairpin structures; the miRNAs are located in the 5' arm of the hairpin structure, whereas the other five are located in the 3' arm of the predicted fold-back structure (Figure 2). Within an miRNA family, the location (organization) of mature miRNA in the fold-back structure is well conserved [40]. Thus, miRNA organization for these two novel miRNAs appears to be atypical.

Changes in expression profile of the species-specific miRNAs were analyzed based on the normalized read abundance in the four different developmental stages. Two novel miRNAs (bmo-mir-2763 and bmo-mir-3001) were present at low levels in larvae relative to other stages (Table 2). The miRNA, bmo-mir-2766 was ubiquitously expressed in all four developmental stages with its highest level found in pupae. Similarly, eight novel miRNAs (sRNA086545, bmo-mir-2733a-2, bmo-mir-2733 g, bmo-mir-2733e-1, bmo-mir-2733f, bmo-mir-2733b, bmo-mir-2733d and bmo-mir-2733 h) were highly expressed in pupae relative to the other stages. Three novel miRNAs (bmo-miR-2998, bmo-mir-2999 and bmo-mir-2763) were most abundantly expressed in the moth stage. Individual counts of the miRNA reads varied considerably, indicating a differential regulation of expression for the novel miRNA family members (Table 2). MicroRNA, bmo-mir-2733a-2 was the most abundantly expressed, followed by bmo-mir-2733 g (Table 2). Taken together, most novel miRNAs were expressed in a stage-specific manner in silkworm. Consistent with these results, genes essential for insect development and metamorphosis are predicted targets for novel miRNAs and miRNA* in silkworm (Additional file 1). We have predicted over 1100 genes as targets for the newly identified silkworm-specific miRNAs and their star species (Additional file 1). These findings suggest an important regulatory role for novel miRNAs in silkworm development.

### Overall miRNA abundance is dynamically regulated during successive developmental stages of silkworm

The proportion of miRNAs to the overall small RNA population varied greatly in different developmental periods. miRNAs accounted for 3.7%, 11.70%, 23.24%, and 15.07% of the total small RNA component from feeding larvae, spinning larvae, pupae and moths, respectively, indicating that overall miRNA abundance changes greatly between any two successive developmental stages of *B. mori*.

The abundance of a given miRNA relative to the overall miRNA abundance in each library was calculated as number of reads of a given miRNA/total number of reads of all miRNAs. Previous reports indicated that in diverse animal species, evolutionarily conserved miRNAs are often highly expressed [38,41,42]. The top three abundant miRNAs of the silkworm are miR-1 (30%), miR-8 (11%) and miR-306 (11%) in the feeding larval stage; miR-1 (40%), miR-8 (18%), and miR-276a (15%) in the spinning larval stage; miR-276a (27%), miR-1 (16%) and miR278 (8%) in the pupal stage; miR-263a (18%), miR276a (16%) and miR-1 (12%) in the adult stage (Additional file 2). Thus, depending on developmental stage, the most abundant miRNA (one individual miRNA) occupies between 18% and 40% of the overall miRNA populations. Furthermore, the top three abundant miRNAs in any given stage cover between 46% and 73% of the overall miRNA populations. This indicates that the most abundantly expressed miRNAs in each stage are different and the levels vary greatly between stages. Interestingly, miR-1 and miR-276a are two of the three most abundant miRNAs in all four stages.

### Temporal regulation of conserved miRNAs during silkworm development

**Figure 4:**
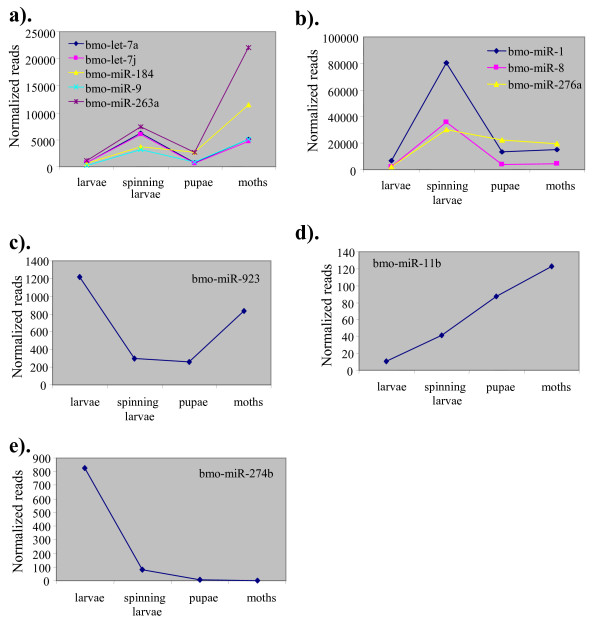
**Temporal regulation of miRNA levels**. (a-e), Plots of expression levels of miRNAs. Normalized read counts in feeding larvae, spinning larvae, pupae and moths are used for the graphical presentation.

In this study, we found evidence for the expression of 101 conserved miRNAs in *B. mori *(Table 3). The relative frequencies of miRNAs generally represent a measure of their expression levels [37,41-43]. We normalized read numbers for all of the miRNAs and compared them on the basis of developmental stages. miRNAs are grouped into following three major categories; The first one is the largest category and with the most dramatic changes in miRNA levels. It includes miR-1, the entire family of miR-2, the miR-9 family (miR-9 and miR-9b), the let-7 family (let-7a, let-7j), miR-10b, miR-31, miR-71, miR-79, miR-87, miR-98, miR-100, miR-252, miR-263a, miR-275, miR-279, miR-317 and miR-1274b (Table 3 and Figure 4a). These miRNAs are at their lowest levels in larvae but greatly increased in abundance during the spinning larval stage and then decreased their levels during the pupal stage but again sharply increased during the adult stage. The second group consists of miRNAs whose abundance in one of the developmental stages is at least twice high as those of the other three. For instance, miR-276a, miR-274b and miR-923 were preferentially expressed in feeding larvae; miR-1, bantam, miR-8, miR-12, miR-98, miR-190, miR-276, miR-305, miR-970 and miR-iab-4 in spinning larvae; miR-9c, miR-14, miR34, miR-92, miR-277, miR-278 and miR-989 in pupae; and miR-31, miR-100, miR-184, miR-263a and miR-275 in adults (Table 3 and Figure 4b). Notably, miR-8 and miR-1 abundance was greater by 10-fold and 6-fold, respectively, in spinning larvae than in other stages. The levels of several miRNAs, such as miR-278 (23-fold), miR-981-1 (7 fold) and miR-9c (5-fold), were greater in pupae relative to other stages. miR-100 (5 fold), miR-31 (3 fold) and miR-184 (3 fold) were expressed at much higher levels in moth relative to other stages. Extreme cases of preferential expression were also observed in the dataset. For instance, miR-274a (11 reads) appeared only in feeding larvae, miR-932 (17 reads) only in spinning larvae, miR-210 (7 reads) and miR-449b (8 reads) only in pupae and miR-1000 (4 reads) and miR-206 (2 reads) only in moths (Table 3). Other types of expression patterns also exist: miR-923 level was moderate in feeding larvae, but decreased in spinning larvae maintained at similar levels in pupae and increased in moths (Figure 4c). While miR-116 level steadily increases during progression of larvae to moth (Figure 4d), miR-274b level gradually decreased during the development (Figure 4e), miR-283 and miR-306 levels were relatively uniform in the four stages (Table 3).

**Table 3:** Normalized reads of conserved miRNAs during different developmental stages of silkworm (TPM: transcripts per million)

**miRNA**	**mature miRNA sequence**	**Feeding larvae**		**Spinning larvae**		**Pupa**		**Moth**	
		**miR**	miR*	**miR**	miR*	**miR**	miR*	**miR**	miR*
bantam	UGAGAUCAUUGUGAAAGCUAAU	168	3	1189	3	210	0	382	6
bmo-let-7a	UGAGGUAGUAGGUUGUAUAGU	817	4	6277	7	748	8	5149	22
let-7b	UGAGGUAGUAGGUUGUGUGGUU	0	0	0	0	0	0	2	0
let-7c	UGAGGUAGUAGGUUGUAUGGUU	7	0	21	0	0	0	63	0
let-7d	AGAGGUAGUAGGUUGCAUAGUU	0	0	0	0	0	0	4	0
let-7e	UGAGGUAGUAGGUUGUUUAGUU	2	0	52	0	2	0	17	0
let-7f	UGAGGUAGUAGAUUGUAUAGUU	0	0	0	0	0	0	2	0
let-7g	UGAGGUAGUAGUUUGUAUAGUU	0	0	17	0	0	0	9	0
let-7j	UGAGGUAGUAGGUUGUAUAGUU	782	0	6042	0	684	0	4674	0
miR-1	UGGAAUGUAAAGAAGUAUGGAG	6947	0	80224	3	13618	0	14873	0
miR-1b	UGGAAUGUUAAGAAGUAUGUA	1	0	7	0	2	0	0	0
miR-2-1	UAUCACAGCCAGCUUUGAUGAGC	48	1	888	0	127	0	1296	0
miR-2-2	UAUCACAGCCAGCUUUGAUGAGC	48	20	888	377	127	400	1296	1309
miR-2b	UAUCACAGCCAGCUUUGAGGAGC	16	0	131	0	8	0	291	0
miR-2c	UAUCACAGCCAGCUUUGAUGGGC	17	0	176	0	12	0	336	0
miR-2d	UAUCACAGCCAGCUUUGUUGAGU	9	0	131	0	18	0	267	0
miR-7	UGGAAGACUAGUGAUUUUGUUGU	294	1	145	0	75	0	405	6
miR-7b	UGGAAGACUAGUGAUUUUUGUU	2	0	0	0	0	0	4	0
miR-8	UAAUACUGUCAGGUAAAGAUGUC	2488	89	35997	321	3738	901	4513	440
miR-9	UCUUUGGUUAUCUAGCUGUAUGA	265	9	3142	245	946	25	5125	878
miR-9b	UCUUUGGUUACCUAGCUGUAUGA	195	0	2357	0	539	0	3879	0
miR-9c	UCUUUGGUAUUCUAGCUGUAGA	0	0	0	0	2	0	4	0
miR-9d	UCUUUGGUAUCCUAGCUGUAG	103	27	456	76	2334	154	457	67
miR-10	UACCCUGUAGAUCCGAAUUUGU	60	248	297	726	389	2047	265	931
mir-10b	CACCCUGUCAGACCAUACUUGUU	123	4	992	7	746	27	1123	2
miR-11	CAUCACAGUCUGAGUUCUUGC	0	0	0	0	0	0	2	0
miR-11b	CAUCACAGUCAGAGUUCUAGCUA	11	3	41	7	87	3	123	4
miR-12	UGAGUAUUACUUCAGGUACUGGU	163	0	453	0	125	0	39	0
miR-13a	UAUCACAGCCACUUUGAUGUG	3	0	14	0	10	2	4	0
miR-13b	UAUCACAGCCAUUUUUGACGAG	28	0	90	0	42	13	347	19
miR-14	UCAGUCUUUUUCUCUCUCCUA	100	1	169	221	651	20	39	9
miR-31	GGCAAGAAGUCGGCAUAGCUG	36	0	1801	0	634	0	5617	2
miR-33	GUGCAUUGUAGUUGCAUUGC	3	14	17	1013	3	43	0	26
miR-34	UGGCAGUGUGGUUAGCUGGUUG	106	0	35	0	3981	8	22	11
miR-71	UGAAAGACAUGGGUAGUGAGAU	2	3	149	62	48	60	86	22
miR-79	UAAAGCUAGAUUACCAAAGCAU	71	2	131	24	35	7	75	9
miR-87	GUGAGCAAACUUUCAGGUGUGU	14	0	200	3	52	18	181	2
miR-92	UAUUGCACCAGUCCCGGCCUA	5	0	38	3	120	2	88	0
mir-92b	AAUUGCACCAAUCCCGGCCU	3	0	14	21	33	0	58	4
miR-98	UGAGGUAGUAGGUUGUAUUGUU	16	0	107	0	17	0	67	0
miR-98 family	UGAGGUUGAAAGUCGCACA	45	0	3	0	3	0	4	0
miR-99	AACCCGUAGAUCCGAGCUUGUU	0	0	3	0	0	0	11	0
miR-100	AACCCGUAGAUCCGAACUUGUG	128	0	636	3	405	0	3223	0
miR-124	UAAGGCACGCGGUGAAUGCCAA	1	0	3	0	0	0	0	0
miR-133	UUUGGUCCCCUUCAACCAGCUG	15	1	55	0	15	0	11	0
miR-137	UAUUGCUUGAGAAUACACGUAG	1	0	7	0	3	0	11	0
miR-183	UAUGGCACUGGUAGAAUUCACU	3	0	0	0	5	0	41	0
miR-184	UGGACGGAGAACUGAUAAGGGC	611	0	3743	3	2660	5	11507	0
miR-190	AGAUAUGUUUGAUAUUCUUGGUUG	22	8	197	55	47	100	34	9
miR-193	UACUGGCCUGCUAAGUCCCAA	2	0	0	0	8	0	0	0
miR-206	UGGAAUGUAAGGAAGUGUGUGG	0	0	0	0	0	0	2	0
miR-210	UGUGCGUGUGACAGCGGCUA	0	0	0	0	7	0	0	0
miR-228	AAUGGCACUGCAUGAAUUCACGG	1	0	17	0	8	0	28	0
miR-236	UAAUACUGUCAGGUAAUGACGCU	3	0	24	0	7	0	2	0
miR-252	CUAAGUACUAGUGCCGCAGGAG	53	0	463	0	224	0	802	0
miR-263a	AAUGGCACUGGAAGAAUUCAC	1177	2	7376	0	2698	0	22066	2
miR-263b	CUUGGCACUGGGAGAAUUCAC	6	1	31	17	108	10	65	17
miR-274	UUUUGUGACCGACACUAACGGGUAAU	11	0	0	0	0	0	0	0
miR-274b	UUUGUGACCGUCACUAACGGGCA	823	29	83	45	5	0	0	0
miR-275	UCAGGUACCUGAAGUAGCGCGCG	32	1	1856	17	215	0	2083	2
miR-276	AGCGAGGUAUAGAGUUCCUACG	26	27	73	73	58	58	15	17
miR-276a	UAGGAACUUCAUACCGUGCUCU	2108	0	30401	0	22507	0	19446	0
miR-276b	UAGGAACUUAAUACCGUGCUCU	1	0	21	0	25	0	22	0
miR-277	UAAAUGCACUAUCUGGUACGACA	111	0	3336	7	6488	47	1815	2
miR-278	UCGGUGGGAUCUUCGUCCGUU	105	78	283	1421	6648	219	37	860
miR-278	UCGGUGGGAUCUUCGUCCGUUU	103	71	280	1269	6546	179	34	737
miR-278	UCGGUGGGAUUUUCGUCCGUUU	2	0	3	0	159	0	2	0
miR-279	UGACUAGAUCCACACUCAU	16	0	408	3	204	5	608	6
miR-279 family	UGACUAGAUUUUCACUUAUCCU	14	12	33	114	15	25	29	63
miR-281	CUGUCAUGGAGUUGCUCUCUUUA	12	303	17	518	65	916	11	188
miR-282	ACCUAGCCUCUCCUUGGCUUUGUCUGU	192	4	577	7	154	2	60	0
miR-283	UAAAUAUCAGCUGGUAAUUCUGGG	222	2	401	0	169	0	237	0
miR-285	UAGCACCAUUCGAAUUCAGUG	1	0	0	0	10	0	0	0
miR-286	GGACUGGAUCCGGACCCGCGUUCUC	1743	0	332	0	200	0	82	0
miR-305	AUUGUACUUCAUCAGGUGCUCUG	39	1	1863	62	28	2	302	45
miR-306	UCAGGUACUAGGUGACUCUGA	2542	3	3256	10	2662	8	4401	4
miR-307	UCACAACCUCCUUGAGUGAG	50	0	944	7	479	0	1332	2
miR-308	AAUCACAGGAUAAUACUGCGAG	134	6	156	31	47	28	50	22
miR-309	UCACUGGGUGCAUGAUGAUCGU	0	0	0	0	327	0	0	0
miR-317	UGAACACAGCUGGUGGUAUCC	117	0	1358	0	671	0	1367	0
miR-429	UAAUACUGUCUGGUAAUGCCG	0	0	3	0	0	0	0	0
miR-449b	AGGCAGUGUUGUUAGCUGGC	0	0	0	0	8	0	0	0
miR-923	GUAAGCGGAGGAAAAGAAACU	1214	0	294	0	255	0	839	0
miR-927	UUUAGAAUUCCUACGCUUUACC	29	30	131	73	75	50	75	58
miR-932	UCAAUUCCGUAGUGCAUUGCAG	0	0	17	0	0	0	0	0
miR-965	UAAGCGUAUAGCUUUUCCCCUU	16	14	21	283	10	135	6	60
miR-970	UCAUAAGACACACGCGGCU	68	0	453	0	202	0	291	0
miR-980	CAGCUGCCUAGCGAAGGGCAA	54	47	20	69	14	23	17	37
miR-981	UUCGUUGUCGUCGAAACCUGCAA	2	0	10	0	30	0	17	0
miR-989-1	UGUGAUGUGACGUAGUGGAA	2	0	477	0	3494	2	45	0
miR-989-2	UGUGAUGUGACGUAGUGGAA	2	0	477	0	3494	0	45	0
miR-993-1	GAAGCUCGUCUCUACAGGUAUCU	0	19	7	169	8	100	6	164
miR-993-2	AAAGCUCGUCUCUACAGGUAUA	0	19	7	176	8	100	6	181
miR-998	UAGCACCAUGGGAUUCAGCUC	12	0	28	0	110	0	54	0
miR-1000	AUAUUGUCCUGUCACAGCAGU	0	0	0	0	0	0	4	0
miR-1175	UGAGAUUCAACUCCUCCAACUUAA	28	189	14	14	2	0	9	0
miR-1274b	UCCCUGUUCGGGCGCCA	3	0	225	0	13	0	304	0
miR-1308	GCAUGGGUGGUUCAGUGG	2	0	0	0	2	0	0	0
miR-iab-4	ACGUAUACUGAAUGUAUCCUGA	17	1	359	3	42	0	6	0
miR-iab-4-3p	CGGUAUACCUUCAGUAUACGUAAC	1	17	3	356	0	43	0	6
miR-iab-4as-5p	UUACGUAUACUGAAGGUAUACCG	1	0	17	0	0	0	0	0

### **miRNAs with extreme low abundance in silkworm libraries**

Several of the conserved miRNA, e.g, miR-124, miR-183, miR-193 and miR-210, were expressed at extremely low levels in different stages (Table 3). This could be due to their restricted expression in specific cell-types. For instance, miR-124 and miR-183 were found in retina of mouse and *Drosophila*, respectively [44-46].

### Differential expression of let-7 family members during development

An advantage of the sequencing-based approach over hybridization-based methods is that changes in expression profile of individual members within a miRNA family can be determined. Our sequence analysis revealed the expression of at least eight members of the let-7 family (let-7a, -7b, -7c, -7d, -7e, -7f, -7 g and -7j) in *B. mori *(Table 3). Spinning larval and adult stages had the highest numbers of reads for let-7a and let-7j. Reads for let-7b, let-7d and let-7f were recovered only in the moth stage, *albeit *in low numbers.

### Expression profile of specific miRNAs differs between *Drosophila*, mosquito and silkworm

MicroRNA, miR-277 expression has been detected only in *Drosophila *adults [20,24]. In contrast, miR-277 is present in the silkmoth at a level much lower than in pupae and spinning larvae (Table 3). In *Anopheles stephensi*, miR-989 is abundantly and specifically expressed in adults [27]. In *B. mori*, the expression of miR-989 was much higher in pupae than in other three stages (Table 3). The miRNA, miR-278 in *Drosophila *is detectable in larvae and increases to peak levels in pupae and adults [47]. In the silkworm, the level of miR-278 was very low in feeding larvae and spinning larvae but increased 23-63 folds in pupae, and then greatly decreased in moths (Table 3). These observations indicate that expression patterns of certain conserved miRNAs differ in insects belonging to different orders taxonomically.

### Accumulation of high-level miR* in different developmental stages

**Figure 5:**
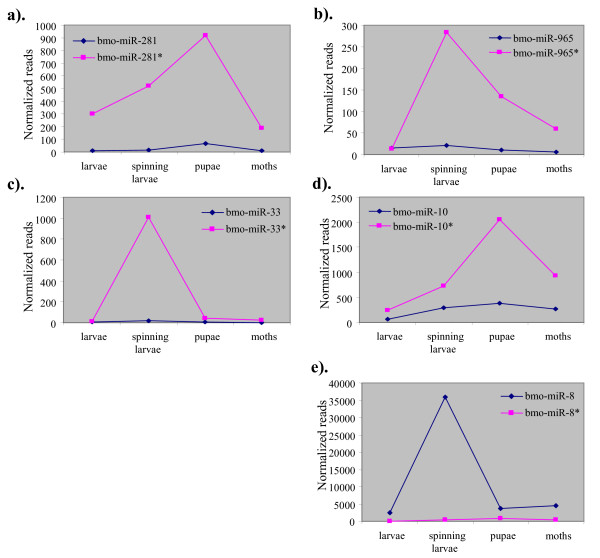
Temporal regulation of miRNA-star (miR*) abundance (a-e), Plots of expression levels of miRNAs along with their miRNA* reads. Normalized read counts in feeding larvae, spinning larvae, pupae and moth stages in silkworm are used for the graphical representation.

Upon processing of a miRNA precursor by Drosha containing complex initially and then by Dicer, the miR:miR* duplex is released, from which only miR is incorporated into RISC. The unincorporated miR* either degrades rapidly or accumulates at significantly low levels [37,38,41,42,48]. Deep sequencing of small RNAs in this study allowed us to monitor the changes in miR* abundance during different developmental stages. Our results largely agree with the previous reports of miR* species recovered at a lower frequency than that of their partners (Table 3 and Figure 5e). However, for several miRNAs i.e, miR-10, miR-33, miR-281 and miR-965, the counts of miR* species far exceeded those of their miR species (Table 3). The relative overall abundance of miR:miR* was in the ratio of 1:48 for miR-33; 1:18 for miR-281; 1:8 for miR-965 and 1:4 for miR-10a (Figure 5a-d). Intriguingly, high level accumulation is strikingly conspicuous during different developmental stages as shown in Figure 5a-d.

### miR-306 and miR-263 loci could generate antisense miRNAs in silkworm

**Figure 6:**
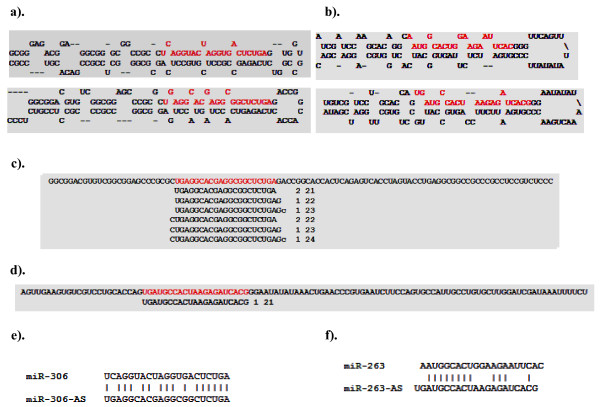
**Antisense miRNAs in *B. mori***. Predicted fold-back of miR-306 (a) and miR-263 (b) from sense (top panel) and antisense strand (bottom panel) and mature miRNA is shown in red. Sequencing reads uniquely aligned to hairpin sequence of reverse complement of miR-306 (c) and miR-263 (d) followed by their cloning frequency and length (nt). Mature sequences of miR-306:miR-306-AS pair (e) and miR-263:miR263-AS (f) aligned for showing identical nucleotide sequences.

Of the two strands of a miRNA locus, in general, one strand (sense strand) is transcriptionally active. Our sequencing efforts identified, for the first time, the prevalence of at least two new miRNA loci, miR-306 and miR-263, that generate miRNAs by convergent transcription from both strands in the silkworm genome. As well, we recovered eighteen reads of miR-iab-4AS, the first antisense miRNA reported in animals [49]. Analysis of the antisense sequences of miR-306-AS and miR-263-AS loci by mFold showed that they can adopt canonical hairpin secondary structures (Figure 6a and 6b). We recovered eight sequence reads that uniquely mapped to the antisense hairpin sequence of miR-306 (miR-306-AS) and a single read mapping to the antisense hairpin of miR-263 (miR-263-AS) (Figure 6c and 6d), which indicates that the antisense transcripts were processed into mature miRNAs *in vivo*. miR-306 is fairly uniformly expressed across the developmental stages, as judged by the number of reads, whereas miR-263 is dynamically regulated from the larva to adult stages. In both cases, the recovery of reads from the antisense strand was much lower than that of the sense strand.

Comparing the mature sequences in each sense and antisense pair showed the seed region in miR-263 and miR263-AS to be almost identical, whereas miR-306 and miR-306-AS showed variations in several internal positions, including the seed region (Figure 6e). In *Drosophila*, miR-iab-4-AS is functional in repressing the Hox family of genes [50,51]. miR-iab-4 and miR-iab-4-AS are not identical in their seed region, so they are predicted to target a different set of genes [51]. The same could be true for miR-306 and miR-306-AS. Identification of three antisense miRNAs for miR-306, miR-263, and miR-iab-4-AS in silkworm suggests that such antisense miRNAs may be more widespread in other organisms.

### Are miR-1920 and miR-1921 genuinely miRNAs in silkworm?

**Figure 7:**
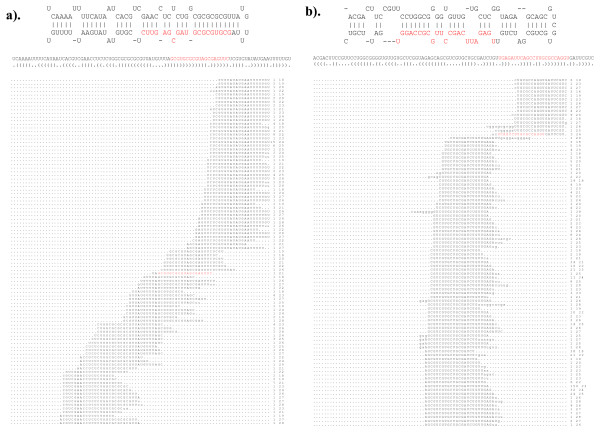
**Overlapping small RNA reads derived from annotated miR-1920 and miR-1921 loci**. Annotated mature miRNAs are shown in red in the predicted fold-back structures for miR-1920 (a) and miR-1921 (b). Overlapping small RNA reads derived from the predicted hairpin structure are shown in the bottom panel and number of reads recovered in the libraries and their length (nt) are shown on the right.

Mapping small RNA reads in this study revealed that certain small RNAs previously annotated as miRNAs may not qualify for such an annotation. In a recent study, two small RNAs were annotated as miR-1920 (one read) and miR-1921 (three reads) in silkworm [35]. Our sequences represented not only the annotated miR-1920 and miR-1921 but also several other small RNAs from these loci. Closer inspection of small RNAs generated from these loci revealed that multiple overlapping small RNA reads could be mapped to almost entire length of the hairpin sequence (Figure 7). These results suggest that the annotated miR-1920 and miR-1921 likely arose from siRNAs generating genomic loci or by fortuitous cleavage of transcripts and not strictly from stem-loop biogenesis. Thus, deep sequencing can aid in confident annotation of small RNAs into miRNAs.

## Discussion

In order to build a normal animal, development of individual tissues and organs needs to be tightly coordinated with the developmental progression of the whole organism [52]. This process is even more critical in insect species, including *B. mori*, that undergo well-defined developmental stages (metamorphosis). To gain insight into post-transcriptional gene regulation in silkworm development, determining a near-complete set of miRNAs and their expression patterns is essential.

### Novel miRNAs in silkworm are preferentially expressed in the pupal stage

Recent studies in Drosophilid species led to the suggestion that most novel miRNAs originated from non-miRNA sequences and only a small fraction (4%) is expected to be retained during evolution [53]. Only 2.5% of the surviving miRNAs are expressed at moderate levels [53]. The observation that most of the newly identified silkworm-specific miRNAs were expressed as abundantly as some of the conserved miRNAs implies that these novel miRNAs evolved, survived, and possibly integrated into the silkworm post-transcriptional regulatory networks. Interestingly, most of these miRNAs showed highest expression in the pupae stage, suggesting a complex gene regulation involving species-specific miRNAs in the development of adult structures.

We have predicted over 1100 genes as targets for the newly identified silkworm-specific miRNAs and their stars using hitsensor software [54] (Additional file 1). Cloning of several novel miRNAs specifically from pupal stage suggests an important role for these miRNAs in larval-pupal-adult metamorphosis. Consistent with this suggestion, many predicted targets for novel miRNAs are genes essential for insect development, molting and metamorphosis, which are regulated by two hormones (ecdysone and its active metabolite 20-hydroxyecdysone (20E) and the sesquiterpenoid juvenile hormone (JH) [55]. A novel miRNA, bmo-mir-2763 is predicted to target diapause hormone receptor-4. *Bombyx *diapause hormone receptor (BmDHR) is expressed in the prothoracic gland (PG), the organ, which synthesizes and releases the insect molting hormones, ecdysteroid [56]. JH synthesis and degradation are two routes that control JH levels in the insect haemolymph (Sheng et al., 2008). Juvenile hormone acid O-methyltransferase (JHAMT) is a key enzyme involved in JH synthesis and is a predicted target for miRNA, bmo-miR-2998. The expression profile of JHAMT mRNA in silkworm and Drosophila melanogaster suggest that the suppression of JHAMT transcript is critical for the induction of larval-pupal metamorphosis [57]. Juvenile hormone esterase (JHE) hydrolyses the JH, thus regulates the levels of JH [58]. JHE is a predicted target for bmo-mir-2766 in silkworm. Thus, the genes encoding enzymes implicated in JH biosynthesis and degradation are predicted targets for novel miRNAs in silkworm. These target predictions suggest that JH levels are regulated by two novel miRNAs that are specifically and abundantly expressed in pupal stage.

A predicted target for novel miRNA (bmo-mir-2763) is nuclear receptor GRF (germ cell nuclear factor (GCNF)-related factor). Increasing evidence suggests that GCNF is important for both female and male reproduction [59]. The developmental program of spermatogenesis is regulated by several transcription factors, one of which appears to be GCNF [59]. GCNF is a sequence-specific repressor of transcription, which binds as a homodimer or an oligomer to its cognate response elements [59]. Identification of GCNF as a target for silkworm-specific miRNA specifically expressed in pupal stage suggests a role for this miRNA in development of male and female reproduction.

During the terminal stages of differentiation of the silkworm ovary, the follicular cells that surround the oocyte produce a large number of related polypeptides that are involved in the formation of the eggshell or chorion [60]. MicroRNA, bmo-mir-2763 is predicted to target a BmGATA beta isoform 3, which regulates the expression of a class of chorion genes expressed during the late stages of choriogenesis [60]. Consistent with the detection of bmo-mir-2763 in the pupal stage, GATA-beta 3 in silkworm has been detected in pupae, but none of the larval tissues [60], suggesting that this process is restricted to pupal stage and novel miRNA (bmo-mir-2763) has a role in the process of choriogenesis. Another miRNA, bmo-mir-2733e-1 is predicted to target NADPH cytochrome P450 reductase, a component of the microsomal P450 electron transport system. It plays an essential role in the transfer of reducing equivalents from NADPH to various P450 molecules and said to be involved on in the ecdysone 20-hydroxylation during the insect's embryonic development [61]

The predicted target for bmo-miR-2998* is silk gland factor-1 (SGF-1). SGF-1 is a member of the fork head/HNF-3 family, which regulates transcription of tissue-specific genes [62]. It plays a role in organogenesis processes such as those of the gut, silk glands, and nervous systems, act as a region-specific homeotic gene [63]. It was also speculated that SGF-1 protein may be initially required for the development of silk glands and subsequently utilized in the control of genes coding for silk proteins. The miRNA, bmo-miR-2998* can potentially target Argonaute-2. Argonaute (Ago) 2 is the catalytic engine of RNA interference, but little is known concerning the regulation of Ago2 by miRNAs or miRNA* in animals, whereas in plants miR168 can target AGO-1, the major Argonaute involved in target mRNA degradation. Recent studies indicate that Ago2 is required for a variety of developmental processes that occur in a tissue-specific manner [64]. These predictions indicate that several of the new miRNAs and miRNA* are likely to play potentially important roles in metamorphosis by regulating genes that participate in these processes.

### Differential regulation of miRNAs during silkworm development implies a role for miRNAs in diverse aspects of *B. mori *physiology and development

Evolutionarily, miR-1 is highly conserved and involved in muscle development and physiology [65,66]. The sole miR-1 gene in *Drosophila *is transcriptionally up-regulated by *Twist *and *Mef2 *during myogenesis [66]. In addition to miR-1, miR-133 and miR-206 are designated as muscle-specific miRNAs because of their abundance in diverse muscle tissues [38,67]. In *B. mori*, miR-1 level is very high, miR-133 is low and miR-206 is extremely low, which suggests that miR-1 likely plays a major role in muscle development and physiology in insects.

Holometabolous insects including silkworm undergoes apoptosis during development from larvae to pupae to moth. The spinning larval stage is a stage of transition to pupae during which gut, salivary glands, the silk gland and larval-specific muscles undergo programmed cell death and histolysis [68]. Several miRNAs known to play a role in apoptosis are expressed in a stage-dependent manner in silkworm. miR-2 and miR-13 families possess identical "seed" sequences, and both show almost similar expression profiles in the silkworm. The miR-2 family is known to target proapoptotic genes (*reaper*, *grim *and *sickle*) in *Drosophila *[69]. Preferential expression of miR-2 and miR-13 families in spinning larvae and moth suggests that these miRNAs may promote apoptosis in these two stages. Another miRNA, miR-34 in *C. elegans*, plays a role in apoptotic and non-apoptotic cell death [70]. In *B. mori*, miR-34 is preferentially and abundantly expressed in pupae relative to the other three stages (Table 3), and this expression profile is similar to what was reported for *C. elegans *[70]. Thus, different miRNAs may play similar roles but in a developmental stage-specific manner in *B. mori*.

Wg/Wnt signaling is highly regulated and inappropriate activation or inhibition of the pathway results in developmental defects and diseases [71,72]. miR-8 is a negative regulator of Wnt signaling in *Drosophila *[73]. Abundant miR-8 during the silkworm spinning-larval stage suggests a role in Wnt signaling. The seed sequences of miR-8 and miR-429 are identical, indicative of a potential in targeting similar sequences (messenger RNA targets). However, in the silkworm, the two miRNAs vary greatly in abundance: miR-8 level was high whereas miR-429 level was extremely low. Perhaps, miR-8 plays a predominant role in Wnt signaling in *B. mori*.

In *Drosophila*, miR-14 and miR-278 are implicated in fat/energy metabolism [37]. miR-278 knockout flies are lean suggesting an excessive utilization of lipid stores. The similarity between the expression profiles of miR-278 in silkworm (Table 3) and *Drosophila *[37] suggests that miR-278 could play a role in regulating energy metabolism in silkworm. miR-14 is also preferentially expressed in pupae of silkworm, which suggests that miR-14 could also play a role in fat metabolism. On the basis of the roles of these two *Drosphila*'s miRNA, it is tempting to speculate that the increased levels of miR-14 and miR-278 observed in the pupal stage could prevent an excessive utilization of fat. This hypothesis would also be consistent with studies in lepidopteran and other insect classes that have shown a preferential utilization of glycogen stores during pupation (Arrese and Soulages, Annual Rev. Entomology, in press). By preventing the utilization of fat, needed for reproduction during the adult stages, these miRNAs would promote the utilization of glycogen stores.

Of all four developmental stages we analyzed, the spinning-larval stage is important - silk is synthesized and a cocoon is built during this period. Strikingly, the overall miRNA abundance is almost doubled from feeding to spinning larval stages, which suggests a role for miRNAs in silk synthesis, besides their importance in developmental transition. The increase in miRNA levels is mostly quantitative and limited to conserved miRNAs. The levels of novel miRNAs in spinning larvae were very low. Similarly, miRNA levels are also sharply elevated in moths relative to the pupae. Again the increase could not be attributed to qualitative differences. Many conserved miRNAs along with a few species-specific miRNAs become more abundant and may regulate reproduction or aging.

The most abundantly expressed miRNAs in B. mori are also highly conserved miRNAs e.g, miR-1, miR-8, miR-10, let-7, miR-263a, miR276a, and miR-306 and were expressed in all four stages, *albeit *their expression levels vary across different developmental periods. These abundantly expressed miRNAs accounted for most of the differences in miRNA abundance in different developmental stages of the silkworm. Variation in the level of a specific miRNA across developmental stages indicates stage-specific function, whereas a similar expression over a range of developmental stages points to a possible role in basal and/or constitutive processes. This analysis also revealed that many miRNAs are similarly regulated during development, suggestive of possible co-regulation of these miRNAs during silkworm development (Figure 4a and 4b). The changes in miRNA expression profile could imply a fine-tuning or tight regulation of their targets in a spatial- and temporal-specific manner. Taken together, conserved miRNAs along with the species-specific miRNAs, in part, orchestrate the developmental progression of *B. mori*.

### miR* levels for several miRNAs are dynamically regulated during development

The specific accumulation of miR* sequences for miR-8, miR-10, miR-37, miR-281 and miR-965 was particularly striking in the silkworm (Table 3 and Figure 5). miR-10* had a higher frequency than miR-10 in *Drososphila *[23,74] and locust [31]. These observations suggest that miR-10* tends to accumulate at a higher level. This implies a potential functional role for miR-10*, and possibly other miR* that tends to accumulate at significant levels. Indeed, Okamura et al. [74] recently demonstrated a regulatory role for miR-276*, which can potentially target a gene and negatively regulate its expression. For several miRNAs, we found that the accumulation of miR* did not correspond with the miR levels in different stages of *B. mori*. miR* accumulation may also be developmentally regulated in the silkworm, which suggests a more complex regulation of miR and miR* sequences than was previously proposed. The observation that several miR*s are more abundant than their corresponding miRNAs indicates that silkworm miR*s could have some endogenous targets. Because of sufficient complementarity between miR and miR* sequences, the accumulation of miR* could potentially regulate the miRNA precursor itself, as was shown for few cases in Arabidopsis [75] and rice (Li and Sunkar, unpublished data).

### Comparision between miRNA array and sequencing-based profiling in the silkworm

Recently, miRNA microarray was used to profile their expression during silkworm metamorphosis [33,36]. The expression patterns of several miRNAs differed between the two previous reports and this study [33,36]. For instance, using miRNA array analysis Zhang et al. [36] reported that miR-9c was highly expressed in larvae, although it was also present in eggs and pupae. This observation prompted the authors to suggest that miR-9c is involved in regulation of metamorphosis from larvae to pupae. However, our sequencing-based analysis of miR-9c abundance sharply contrasts with that in the previous report (Table 3 Figure 4a). First, miR-9 family represented by three members, miR-9, miR-9b and miR-9c in silkworm and these members differ by one nucleotide. Thus, it is difficult to determine which of these members is being detected from a hybridization-based approach. Second, miR-9c expression is extremely low: we recovered one read from the pupae and two reads from the adults, and none from the feeding or spinning larvae (Table 3). Third, we recovered abundant miR-9 and miR-9b reads from all four libraries, and both exhibited a dynamic regulation (Figure 4). Overall, miR-9b level was lower than that of miR-9 in any given stage. miR-9 family had the highest expression in moths and the lowest in larvae. In another miRNA array-based study, He et al., [33] demonstrated that let-7b and let-7d were expressed only in larval and pupal stages but not in the adult stage. Since let-7 family is represented by eight members in the silkworm, each of which differed by one nucleotide, which makes distinguishing difficult by the hybridization-based approach. On the basis of read counts, only let-7a and let-7j are highly abundant, and the remaining six members are low (Table 3). In contrast to what was reported [33], we recovered reads for both let-7b and let-7d from the adult library (*albeit *in low numbers) but none from the larval or pupal libraries. Another miRNA, miR-277, was reported to be specifically expressed in the moth stage [33]. We detected miR-277 in all four stages. The level was highest in pupae and estimated to be threefold greater than that in the moths. Similarly, the expression profiles of several other miRNAs, as deduced from the array analysis, did not match our analysis. Perhaps, the array-based approach [33,36] needs further validation and we believe that the sequencing-based study is more reliable. Results from miRNA array analysis are likely affected by false signals, cross-hybridizations or background noise.

## Conclusions

Deep sequencing of small RNAs from four distinct developmental stages of the silkworm (feeding larvae, spinning larvae, pupae and moths), each with distinct anatomy, morphology and physiology, allowed us to comprehend changes in the miRNA expression profiles during development and to discover novel miRNAs in *B. mori*. Our study represents the first exhaustive expression profiling of miRNAs in the major developmental stages. We discovered 14 novel miRNAs in the silkworm that are potentially species-specific, identified 101 conserved miRNAs, monitored the dynamic changes in miRNA levels during metamorphosis, and identified miR* species that accumulate at higher levels than their corresponding miRNAs. The accumulation of miR*appears to be regulated in a stage-specific manner. In addition, we found novel antisense miRNA loci in silkworm. Future identification of the miRNA targets should enable us to integrate the data into post-transcriptional regulatory networks important for silkworm metamorphosis, as well as other economically important traits such as silk synthesis.

## Methods

### Insect rearing, total RNA preparation, and miRNA library construction

*B. mori *eggs and diet were purchased from Carolina Biological Supply (Burlington, NC) and larvae were reared at ambient temperature. Whole insects in the feeding larval (4th and 5th instars), spinning larval, pupal, and adult stages were separately frozen in liquid nitrogen and grinded for total RNA extraction using Trizol reagent (Invitrogen Life Technologies, Carlsbad, CA). Total RNA was dissolved in de-ionized formamide for effective RNA denaturation. Small RNAs in the desired size range (15-30 nt) were purified from denaturing 15% polyacrylamide gel and sequentially ligated with the 3' and 5' adapters as described previously [38]. Reverse transcription reaction was performed using the RT primer (5'CAAGCAGAAGACGGCATACGA), and the forward and reverse primers (5'AATGATACGGCGACCACCGACAGGTTCAGAGTTCTACAGTCCGA and 5'CAAGCAGAAGACGGCATACGA) primers. After phenol/chloroform extraction and ethanol precipitation, the PCR products were shipped to Illumina (city, CA) for sequencing.

### Sequence analysis and identification of homologs of conserved miRNAs in *B. mori*

Our computational methods for analyzing the small RNA library was reported previously [38]. In brief, all small RNA reads without perfect matches to the most proximal 11 nt of the 5' adaptor sequences were first removed. Reads corresponding to repeats were discarded using the Einverted and Etandem programs in the EMBOSS package. The unique small RNAs were aligned to RepBase v13.04 [76] and known non-coding RNAs (rRNAs, tRNAs, snRNAs, snoRNAs, etc.) obtained from Rfam [77] with NCBI BLASTN. After removal of repetitive elements, the remaining small RNAs were mapped to the messenger RNAs and such sequences were discarded assuming that these represent degradation products. The filtered sequences were mapped to the reported miRNAs in the miRBase v13. Small RNAs that matched known miRNAs of the silkworm or other animal species resulted in identification of conserved miRNA homologs in *B. mori*.

### Identification of novel miRNAs in *B. mori*

The remaining unique small RNAs were aligned to genome sequences of *B. mori *downloaded from Silkworm Genome Database [78] and for those sequences that matched with the genome, the fold-back structures were predicted using their flanking sequences [79]. Candidates with predictable fold-back structures were analyzed for the presence of miR* sequences in library. Initially, for such candidates we predicted miR* sequences with 2 nt 3' overhangs on the opposite arm of the fold-back structures and subsequently we searched for the presence of such miR* sequences in our library. This resulted in identification of 12 miRNAs. Additional two small RNAs (bmo-mir-2733e-1 and bmo-mir-2733f) without their miR* were added to the list of new miRNAs, because these two small RNAs are members of the newly identified miRNA family in *B. mori*.

## Authors' contributions

R.S. designed the research, analyzed the data, coordinated the project and wrote the paper. G.J. constructed the small RNA library. Y.Z. and W.Z. performed the computational analysis. N.S and H.J. reared the insects and isolated total RNA used for library construction. H.J. also edited the manuscript. J.L.S. and E.A helped with the analysis and interpretation of the computational dataset. All authors read and approved the final manuscript.

## Supplementary Material

Additional file 1 Predicted targets for the newly identified miRNAs and miRNA*s in silkworm. For each predicted target, accession number, annotation and hit scores are provided. Click here for file

Additional file 2 **Top 20 most abundantly expressed miRNAs in silkworm during different developmental stages**. Proportion of reads recovered for miRNAs in each of the four developmental stages, i.e., feeding larvae, spinning larvae, pupae and moth are provided. Click here for file
