# Supplementary material for: Deep sequencing of small RNA libraries reveals dynamic regulation of conserved and novel microRNAs and microRNA-stars during silkworm development
Source: BMC Genomics. 2010 Jan 20;11:52. doi: 10.1186/1471-2164-11-52 (PMC2824724; doi:10.1186/1471-2164-11-52)
Supplement: Additional file 2 — Top 20 most abundantly expressed miRNAs in silkworm during different developmental stages. Proportion of reads recovered for miRNAs in each of the four developmental stages, i.e., feeding larvae, spinning larvae, pupae and moth are provided. [file 1471-2164-11-52-S2.PPT]

## Slide 1
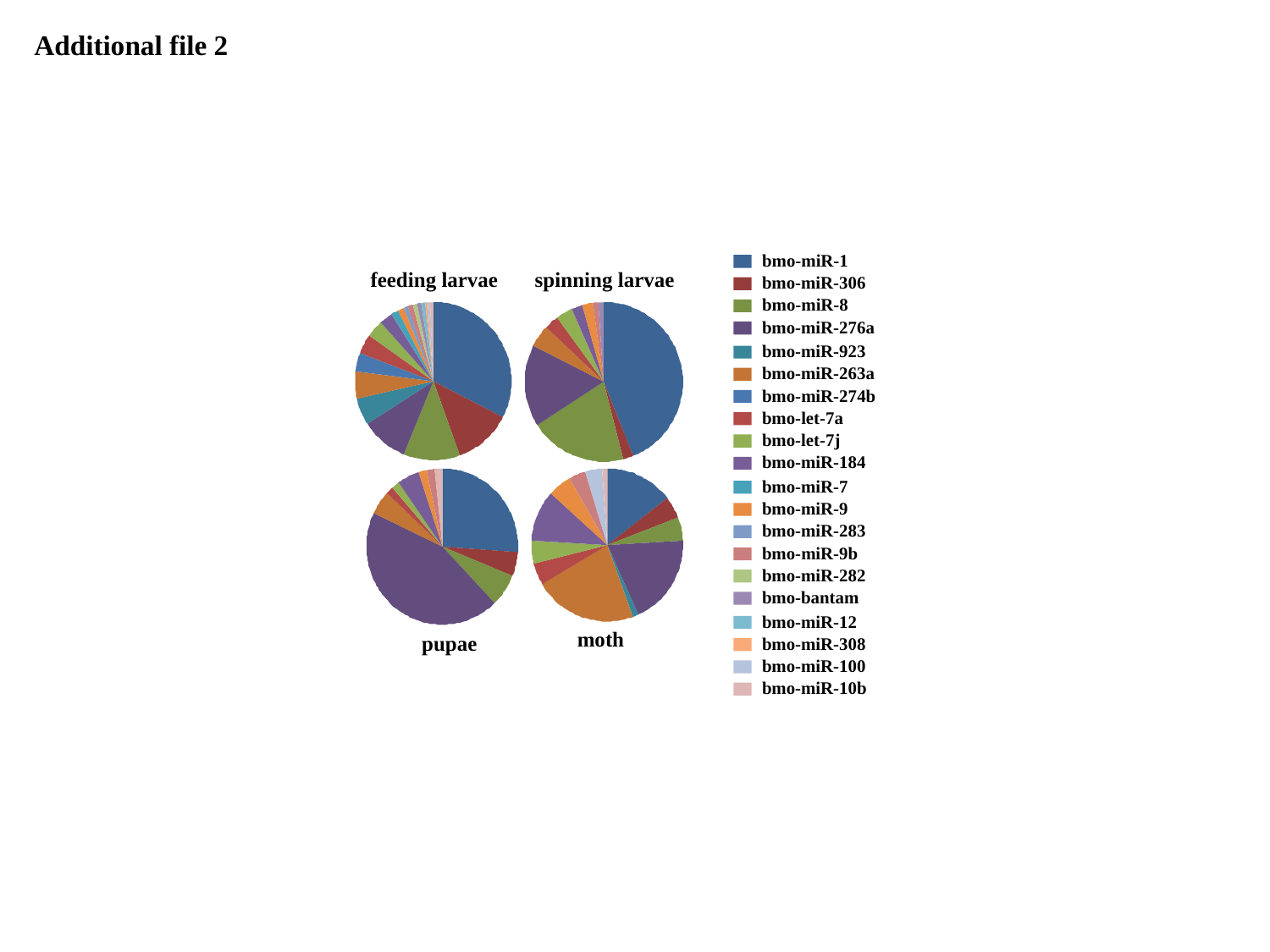

Additional file 2
bmo-miR-1
bmo-miR-306
bmo-miR-8
bmo-miR-276a
bmo-miR-923
bmo-miR-263a
bmo-miR-274b
bmo-let-7a
bmo-let-7j
bmo-miR-184
bmo-miR-7
bmo-miR-9
bmo-miR-283
bmo-miR-9b
bmo-miR-282
bmo-bantam
bmo-miR-12
bmo-miR-308
bmo-miR-100
bmo-miR-10b
feeding larvae
spinning larvae
moth
pupae
